# Restriction Enzyme Based Enriched L1Hs Sequencing (REBELseq): A Scalable Technique for Detection of Ta Subfamily L1Hs in the Human Genome

**DOI:** 10.1534/g3.119.400613

**Published:** 2020-03-04

**Authors:** Benjamin C. Reiner, Glenn A. Doyle, Andrew E. Weller, Rachel N. Levinson, Esin Namoglu, Alicia Pigeon, Emilie Dávila Perea, Cynthia Shannon Weickert, Gustavo Turecki, Deborah C. Mash, Richard C. Crist, Wade H. Berrettini

**Affiliations:** * Center for Neurobiology and Behavior, Department of Psychiatry, Perelman School of Medicine, University of Pennsylvania, Philadelphia, Pennsylvania, 19104; † Schizophrenia Research Laboratory, Neuroscience Research Australia, Randwick NSW 2031, Australia; ‡ School of Psychiatry, Faculty of Medicine, University of New South Wales, Sydney NSW 2052, Australia; § Department of Neuroscience & Physiology, Upstate Medical University, Syracuse, New York 13210; ** McGill Group for Suicide Studies, Douglas Mental Health University Institute, McGill University, Montreal, QC H4H 1R3, Canada; †† Dr. Kiran C. Patel College of Allopathic Medicine, NOVA Southeastern University, Fort Lauderdale, Florida, 33314

**Keywords:** REBELseq, Retrotransposons, L1, L1Hs

## Abstract

Long interspersed element-1 retrotransposons (LINE-1 or L1) are ∼6 kb mobile DNA elements implicated in the origins of many Mendelian and complex diseases. The actively retrotransposing L1s are mostly limited to the L1 human specific (L1Hs) transcriptional active (Ta) subfamily. In this manuscript, we present REBELseq as a method for the construction of Ta subfamily L1Hs-enriched next-generation sequencing libraries and bioinformatic identification. REBELseq was performed on DNA isolated from NeuN+ neuronal nuclei from postmortem brain samples of 177 individuals and empirically-driven bioinformatic and experimental cutoffs were established. Putative L1Hs insertions passing bioinformatics cutoffs were experimentally validated. REBELseq reliably identified both known and novel Ta subfamily L1Hs insertions distributed throughout the genome. Differences in the proportion of individuals possessing a given reference or non-reference retrotransposon insertion were identified. We conclude that REBELseq is an unbiased, whole genome approach to the amplification and detection of Ta subfamily L1Hs retrotransposons.

Retrotransposons are a class of mobile DNA elements capable of replicating and inserting copies of themselves elsewhere in the genome using an RNA intermediate (Richardson *et al.* 2014). Long interspersed element-1 (L1), a type of retrotransposon that is currently active in the human genome, is estimated to constitute approximately 17% of the human genome (Beck *et al.* 2010). Despite their abundance, most L1s are truncated or mutated to the point of no longer being able to retrotranspose (Ostertag and Kazazian 2001). There are ∼100 L1s in each human genome that are competent, meaning they are full length and capable of replicating, the vast majority of which belong to the L1 human specific (L1Hs) Ta subfamily (Boissinot *et al.* 2000). Alternatively, and in agreement with Kazazian *et al.* (1988), Beck *et al.* described two competent L1s of the pre-Ta subfamily (Beck *et al.* 2010). A competent L1Hs is ∼6 kb in length and contains a promoter, 5′ and 3′ untranslated regions, and two open reading frames (ORF): ORF1, encoding an RNA binding protein (Naufer *et al.* 2016), and ORF2, encoding a fusion protein that acts as both a reverse transcriptase (Mathias *et al.* 1991) and endonuclease (Feng *et al.* 1996).

Whereas competent retrotransposons are mostly limited to full length L1Hs of the Ta subfamily, the remnants of vast numbers of retrotransposons, from both evolutionarily older and Ta subfamily L1Hs can be found throughout the human genome. Notably, Ta subfamily L1Hs contain identifying nucleotides in the 3′ UTR that can be utilized for their differential amplification by polymerase chain reaction (PCR) (Boissinot *et al.* 2000). Retrotransposition of competent L1Hs elements frequently results in 5′ truncation of the new Ta subfamily L1Hs element insertions. Therefore, to reliably amplify and detect full length and truncated Ta subfamily L1Hs elements, including germline polymorphic and individual somatic mutations, PCR primers for amplification must be targeted near the 3′ end of the L1Hs Ta subfamily sequence.

In this manuscript, we present REBELseq (Restriction Enzyme Based Enriched L1Hs sequencing), a scalable technique for the differential amplification of both full length and 5′ truncated L1s. As described below, we specifically target the Ta subfamily of L1Hs and summarize the results of the application of REBELseq to DNA samples from 177 individuals.

## Materials And Methods

### Brain samples

177 fresh frozen human postmortem prefrontal cortex brain samples (Brodmann’s Area 9 or 10) were provided by the Douglas-Bell Canada Brain Bank at McGill University, the Human Brain and Spinal Fluid Resource Center at UCLA, the New South Wales Brain Tissue Resource Center or the University of Miami Brain Endowment Bank. All work was approved by the University of Pennsylvania Institutional Review Board as category IV exempt human subject research. All reported age, sex and ethnicity data are based on associated medical records. The sample set contained 138 males (Age 45.6 ± 15.5; 93 European, 25 African, 15 Hispanic, 2 Asian and 3 of unknown ethnic origin) and 39 females (Age 55.5 ± 19.2; 33 European, 1 African, 3 Hispanic and 2 of unknown ethnic origin). All samples were from sudden death cases without prolonged agonal conditions. Causes of death involving neural trauma or asphyxiation and post-mortem intervals > 48 hr were excluded.

### Purification of genomic DNA from NeuN+ nuclei

Brain samples were processed and stained for NeuN by modification of a previously described method (Jiang *et al.* 2008). Frozen brain samples (40-80 mg) were homogenized on wet ice using a Dounce homogenizer (Sigma #D8938-1SET) in 1 mL sucrose lysis buffer, containing 0.32 M sucrose, 5 mM CaCl_2_, 3 mM MgOAc_2_, 0.1 mM EDTA, 10 mM Tris, 1 mM DTT, 1 mM PMSF, 15 µL protease inhibitor cocktail (Sigma #P8340), 5 µL RNaseOUT (Invitrogen #10777019) and 1 µL Trition X-100, using 25 strokes with the large clearance pestle and 25 strokes with the small clearance pestle. 500 µL aliquots of homogenate were layered on top of 200 µL of sucrose cushion solution, containing 1.8 M sucrose, 3 mM MgOAc_2_, 10 mM Tris, 1 mM DTT, 1 mM PMSF, 2 µL protease inhibitor cocktail and 2.5 µL RNaseOUT, in a 1.5 mL Eppendorf style tube and centrifuged at 21,130 × g for 30 min (min) at 4° to pellet nuclei. The middle protein layer and upper liquid phase, containing total cytosolic RNA, were removed, diluted in 4 mL of TRIzol solution (Invitrogen #15596-018) and frozen at -80° for future analysis. The remaining sucrose cushion solution was removed and discarded, taking care not to disturb the nuclei pellet. 65 µL of PBS + 3 mM MgCl_2_ was added to each nuclei pellet and incubated on wet ice for 15 min, to allow the pellet to soften. Following incubation, pellets were further loosened by gentle up and down pipetting. The solutions containing each pair of pellets, from the previously separated homogenate, were transferred to a 2 mL Eppendorf style tube for nuclei staining, with 55 µL PBS + 3 mM MgCl_2_, 25 µL BSA blocking solution, containing 0.5% BSA and 10% normal goat serum, 10 µL 7.5 µM DAPI and 30 µL 1:10 diluted α-NeuN-AF488 antibody (Millipore #MAB377X). Nuclei staining reactions were incubated on a nutator, protected from light, at 4° for 60 min. Staining solutions were loaded onto Falcon tubes with 30 µm cell strainer caps (Fisher #08-771-23) and filtered by gravity flow.

**Figure 1:**
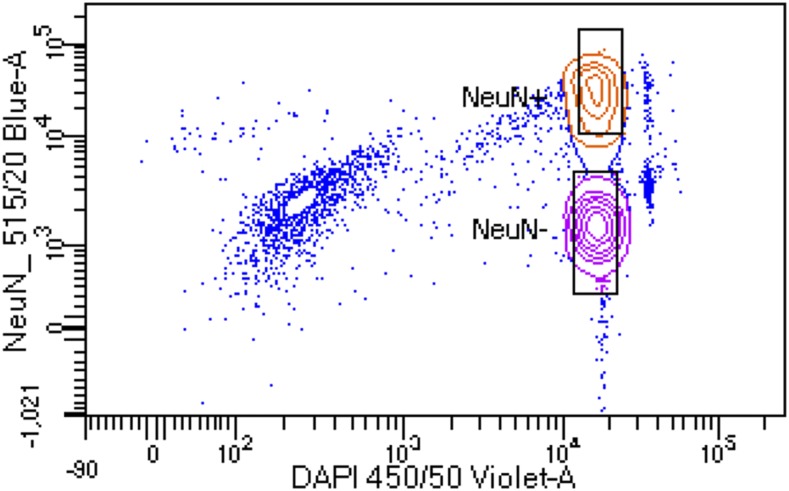
Florescence assisted cell sorting (FACS) of NeuN stained nuclei. Representative image showing FACS separation of NeuN+ and NeuN- DAPI stained nuclei. Boxes indicate gating for populations of nuclei that were collecting for gDNA isolation. Only NeuN+ gDNA was utilized for this work.

Labeled nuclei were sorted into NeuN+ and NeuN- fractions in 15 mL conical vials containing 500 µL of PBS + 3 mM MgCl_2_ cushion using a FACSAria II (Beckman-Coulter; Figure 1). After sorting, NeuN+ and NeuN- nuclei fractions were diluted to 2 mL final volume using PBS + 3 mM MgCl_2_. 400 µL (0.2 volumes) of sucrose cushion solution, described above, was added to each tube, mixed by gentle inversions and allowed to incubate, on ice, for 15 min. Diluted nuclei fractions were centrifuged at 2,000 × g for 30 min at 4° to pellet nuclei, after which, all but 100 µL of supernatant was removed by careful pipetting. 600 µL digestion buffer, containing 50 mM Tris, 100 mM EDTA, 100 mM NaCl and 1% SDS, and 35 µL proteinase K solution (Sigma #3115828001) was added, mixed by vortexing and allowed to incubate, overnight, in a 56° water bath. The following morning, NeuN+ and NeuN- genomic DNA (gDNA) were isolated using the Zymo Research genomic DNA clean and concentrator kit (#D4011), utilizing the manufacturer’s protocol, and the concentration was determined using a Qubit 3 fluorometer and high-sensitivity double-stranded DNA assay (ThermoFisher #Q32854). The NeuN+ gDNA was utilized for this study and the NeuN- gDNA was saved for future use.

### Ta subfamily L1Hs-enriched library construction and sequencing

**Figure 2:**
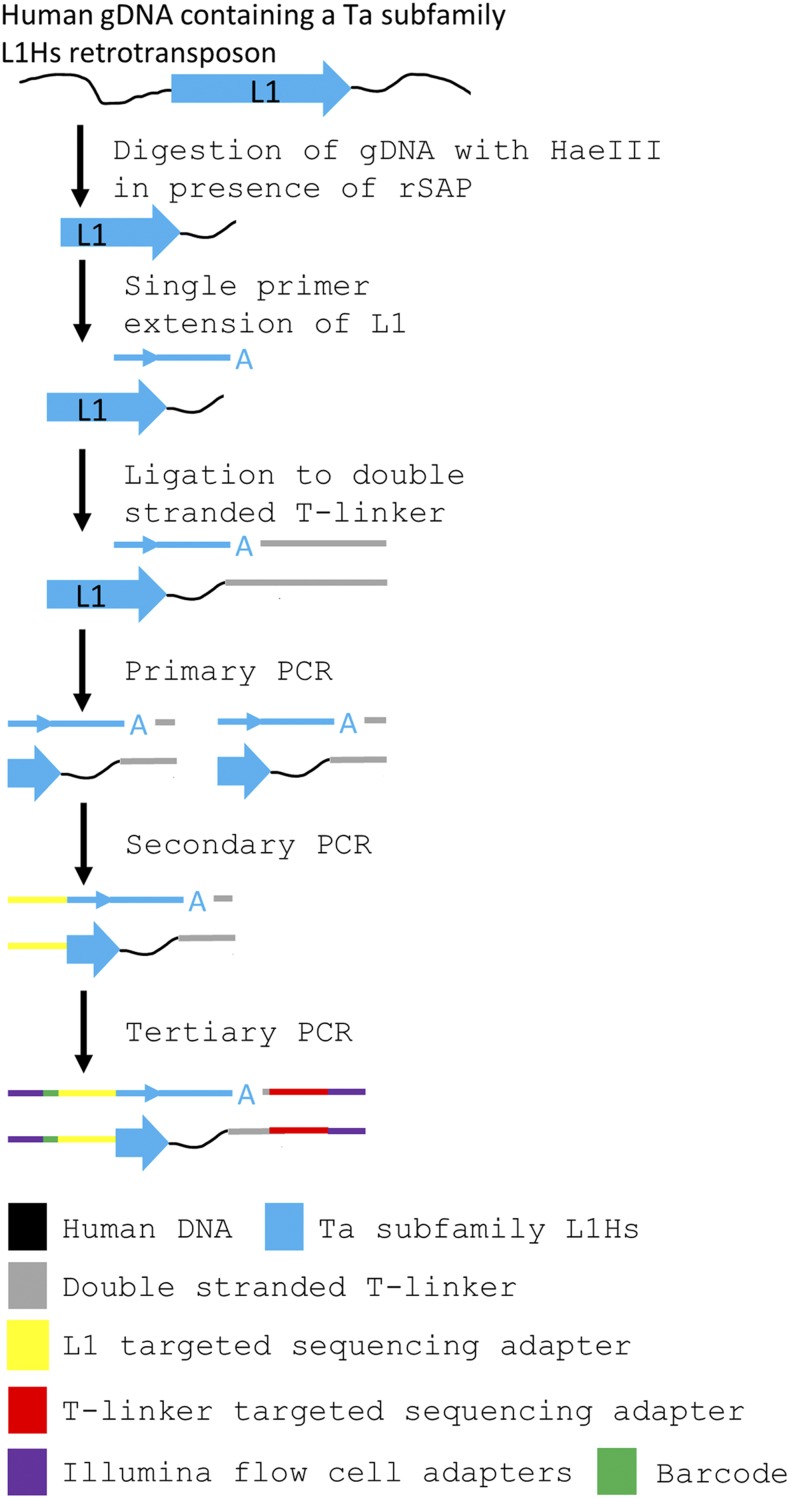
Schematic of the construction of Ta subfamily enriched L1Hs sequencing libraries. gDNA isolated from NeuN+ nuclei was enzymatically digested with HaeIII in the presence of shrimp alkaline phosphatase (rSAP) to fragment the genome and remove 5′ phosphates from cleavage products. A single primer extension using the Ta subfamily specific L1HsACA primer extends the 3′ end of the L1 sequence into the downstream gDNA. The 3′ ‘A’ overhang from the single primer extension is ligated to a custom T-linker, and primary PCR amplifies the construct using L1HsACA and T-linker specific primers. Hemi-nested secondary PCR using the L1Hs specific L1HsG primer and T-linker primer reduces the length of the L1 sequence carried forward and adds a sequencing adapter to the L1 end. Tertiary PCR uses primers complementary to the 5′ end of library amplicons to add a barcode to the L1 end and Illumina flow cell adapters to both ends of the amplicons.

Ta subfamily L1Hs-enriched next generation sequencing libraries were constructed utilizing the REBELseq technique (Figure 2). 33 ng (∼5000 diploid genomes) of NeuN+ gDNA for each sample was digested with 5 U HaeIII (New England Biolabs #R0108M) in a 10 µL reaction, containing 1X CutSmart buffer (New England Biolabs #B7204S) and 1 U recombinant shrimp alkaline phosphatase (to remove 5′ phosphates; New England Biolabs #M0371S) according to the manufacturer’s protocol (HaeIII digestion, Figure 2). Samples were incubated at 37° for 60:00 min, then 80° for 20:00 min, then a 4° hold. All the digested NeuN+ gDNA was then used as a template for a 25 µL single cycle primer extension reaction (Single Primer Extension, Figure 2), containing 1X GoTaq HotStart Colorless Master Mix (Promega #M5133) and 33.2 fmol/µL L1HsACA primer (See Supplemental Oligomers), which should extend only Ta subfamily L1 3′ UTR sequence (Boissinot *et al.* 2000) and the adjacent downstream genomic DNA. Samples were incubated at 95° for 2:30 min, then 95° for 0:30 min, 60° for 1:00 min, 72° for 1:00 min, then a 4° hold. All of the L1HsACA primed, 3′ A-overhang extension products were ligated to a double stranded T-linker molecule (See Supplemental Oligomers), with a 5′ phosphate on the top strand, in a 30 µL reaction (T-linker ligation, Figure 2), containing 1X T4 DNA Ligase Buffer (New England Biolabs #B0202S), 400 U T4 DNA Ligase (New England Biolabs #M0202L) and 2.78 pmol/µL double stranded T-linker. Samples were incubated at 16° for 120 min, then 65° for 20 min, then a 4° hold. All of the T-linker ligated, Ta subfamily L1 elements were amplified (Primary PCR, Figure 2) to enrich the number of copies of each unique Ta subfamily L1 in a 50 µL reaction, containing 1X GoTaq HotStart Colorless Master Mix, 0.2 pmol/µL L1HsACA primer and 0.2 pmol/µL T-linker bottom strand primer (See Supplemental Oligomers). Primary PCR was thermally cycled as 95° for 2:30 min, then 20 cycles of 95° for 0:30 min, 60° for 0:30 min, 72° for 1:30 min, then 72° for 5:00 min, and finally a 4° hold. The primary PCR product was then diluted 1:10 in nuclease free water and used as template in a 25 µL hemi-nested PCR reaction (Secondary PCR, Figure 2), containing 2.5 µL 1:10 diluted Primary PCR, 1X GoTaq HotStart Colorless Master Mix, 0.2 pmol/µL T-linker bottom strand primer and 0.2 pmol/µL Seq2-L1HsG primer (See Supplemental Oligomers). Secondary PCR was thermally cycled as 95° for 2:30 min, then 35 cycles of 95° for 0:30 min, 60° for 0:30 min, 72° for 1:30 min, then 72° for 5:00 min, and finally a 4° hold. The secondary PCR product was cleaned and size selected using KAPA Pure Beads (Roche #KK8002) according to the manufacturer’s protocol for a 0.55X-0.75X double-sided size selection, generating a 12 µL eluate of purified library of 200-1,000 bp fragments. The purified secondary PCR library was then used as a template for the addition of one of six single indexed Illumina flow cell adapters in a 50 µL reaction (Tertiary PCR, Figure 2), containing 11 µL purified secondary PCR library, 1X Phusion HF Polymerase Buffer (New England Biolabs #B0518S), 2.5 U Phusion Hot Start HF polymerase (New England Biolabs #M0535L), 0.2 mM dNTPs (Thermo #R0191), 0.5 pmol/µL Adapt2-Seq1 primer and 0.5 pmol/µL Adapt1-Barcode-Seq2 primer (See Supplemental Oligomers). Tertiary PCR was thermally cycled as 98° for 0:30 min, then 15 cycles of 98° for 0:05 min, 72° for 0:15 min, then 72° for 1:00 min, and finally a 4° hold. Finally, the tertiary PCR reactions underwent final cleanup and size selection by KAPA Pure Beads 0.55X-0.75X double-sided size selection, with a final eluant volume of 22 µL. This purification was verified, and the average fragment size of the library was determined, using the 2100 Bioanalyzer and high sensitivity DNA kit (Agilent #5067-4626; Supplemental Figure 1).

The barcoded, L1-enriched sequencing libraries were quantified, in triplicate, for the concentration of PCR fragments properly flanked by the Illumina flow cell adapters using the KAPA Library Quantification Kit (Roche #KK4835) and 7900HT real-time PCR (Applied Biosystems) according to the manufacturer’s protocol, utilizing 1:20,000, 1:200,000 and 1:2,000,000 dilutions. Using the KAPA Library Quantification Analysis Template_ILM_v4-1, the Ct values and the average fragment size (determined above by Bioanalyzer), the concentration of sequenceable library was calculated for each sample. Samples were pooled in groups of six, each having a unique barcode from the Adapt1-Barcode-Seq2 primer (described above), in 30 µL pools of 25 nM equimolar final concentration. Pooled libraries were then sequenced by the University of Pennsylvania Next-Generation Sequencing Core, using one pool per lane, on an Illumina HiSeq 4000 utilizing 150 bp paired-end sequencing and 10% PhiX sequencing control spike-in (Illumina #FC-110-3001). Raw sequencing data were evaluated by Illumina quality filters and reads passing filter were deconvoluted by sequencing lane and barcode and transferred into read 1 and read 2 (paired end) FASTQ files.

### Bioinformatics

Demultiplexed FASTQ files from sequencing were transferred to the Penn Medicine Academic Computing Services servers for analysis. Initial evaluation of sequencing read quality showed high quality reads for read 1 and diminished quality for read 2 (paired end reads), likely due to the need to sequence through the low diversity L1HsG primer and the low complexity, highly repetitive, variable length poly(A) tail of L1Hs elements. Therefore, only read 1 sequence data were used for all subsequent bioinformatics. PhiX spike-in sequences were removed from an individual’s FASTQ file using BBTools (https://sourceforge.net/projects/bbmap) bbduk (BBTools decontamination using k-mers) and the PhiX sequence as reference. Remaining reads were end trimmed of Illumina flow cell and sequencing adapters, and cleaned based on a Phred quality score of Q ≥ 20 using bbduk. Trimmed reads were aligned to the hg19 build of the human genome with Bowtie2-2.1.0 (Langmead and Salzberg 2012) using the end-to-end, very sensitive alignment parameters, to generate a sequence alignment map (SAM) file for each individual. Investigation into the unaligned reads showed that ∼10% contained a poly(T) stretch after the genomic portion of the read that was unalignable, possibly corresponding to the poly(A) sequence at the 3′ end of non-reference L1Hs elements. As such, bbduk was used to k-trim poly(T) stretches from the 3′ end of the unaligned reads using a k-mer of 11 T’s and a hamming distance of 1. The poly(T) trimmed reads were realigned to hg19 with Bowtie2-2.1.0 using end-to-end, very sensitive alignment parameters, generating a second SAM file for each individual. Each SAM file was converted to a binary alignment map (BAM) format, during which alignments with a MapQ score < 30 were removed using samtools-0.1.19. The two BAM files for each individual, one from each alignment, were merged, sorted and indexed using samtools-0.1.19. Using samtools-0.1.19, BAM files from all individuals were merged into a single BAM file using RG tags (-r option), and this combined BAM file was sorted and indexed. The single merged BAM file with RG tags was split into BAM files containing the data for each chromosome using samtools-0.1.19, and the chromosome specific BAM files were used as input for the custom python script REBELseq_v1.0. REBELseq_v1.0 utilizes the sorted and indexed reads in the chromosome specific BAM files to generate peaks of overlapping reads within a 150 base pair sliding window, and does so with respect to DNA strand. Each peak corresponds to a putative L1 retrotransposon insertion, for which the peak’s genomic coordinates, number of unique reads per peak, number of reads per individual sample and average read alignment quality (mean MapQ) are determined. The REBELseq_v1.0 output file was then further annotated using the custom python script REBELannotate_v1.0 for L1Hs annotated in hg19 repeat masker and L1Hs identified in the 1000 genomes data (Sudmant *et al.* 2015). REBELannotate_v1.0 utilizes a browser extensible data (BED) formatted file to annotate genomic features of interest that overlap with or occur within 500 base pairs downstream, with respect to strand, of the peak being annotated. All REBELseq_v1.0 peaks that were annotated by REBELannotate_v1.0 as an L1Hs in hg19 repeat masker are referred to throughout the manuscript as reference L1Hs. All peaks that did not annotate to an hg19 repeat masker L1Hs are collectively referred to as non-reference L1Hs. Non-reference L1Hs that annotate to the 1000 genomes data are referred to as known non-reference L1Hs and non-reference L1Hs that did not annotate to the 1000 genomes data are putatively referred to as novel non-reference L1Hs. The novel non-reference L1Hs include what are likely real L1Hs insertions (because they meet our cutoff criteria) that inserted into older repetitive elements (Alu, L1Pa, etc.) within the reference genome. The REBELseq_v1.0 and REBELannotate_v1.0 custom scripts are based on work originally described by Ewing and Kazazian (Ewing and Kazazian 2011), and all python scripts and necessary reference files discussed in this manuscript are available online (https://github.com/BenReiner/REBELseq). Scrutiny of the output data from REBELseq showed a low level of index hopping between multiplexed samples. Index hopping was remediated by removing samples with less than 6% (Costello *et al.* 2018) of the maximum read count per set of six multiplexed samples for a given L1 insertion. It is notable that index hopping could be eliminated by utilizing a second unique bar code in tertiary PCR (*i.e.*, unique dual-indexing), thereby causing index hopped reads to be discarded during demultiplexing (MacConaill *et al.* 2018).

### PCR validations

Primers used in PCR experiments for method validation were designed using Primer3-2.2.3 in a Perl script (makeprimers.pl), originally written by Adam Ewing (Ewing and Kazazian 2011), with an optimal Tm setting of 58° (minimum 56° and max 64°), an optimal primer length of 24 nucleotides (minimum 21 and max 27) and the GC-clamp option set to 1. 25 µL reactions were constructed using 1x Go-Taq colorless hotstart master mix (#M5133, Promega), 1 ng of gDNA for both an individual predicted to have a particular L1Hs insertion or an individual predicted not to have the insertion, and either 0.2 µM of the filled site and empty site (empty allele detection) or the filled site and L1HsACA primer (L1 filled allele detection). Samples were thermally cycled as 95° for 2:30 min, then 10 cycles of touchdown PCR at 95° for 0:30 min, 69-60° for 0:30 min, 72° for 1:30 min, then 25 cycles of 95° for 0:30 min, 60° for 0:30 min, 72° for 1:30 min, then 72° for 5:00 min, and finally a 4° hold. Go-Taq green flexi buffer (#M8911, Promega) was added to all samples after amplification, and samples were separated by 1.2% gel electrophoresis and visualized on a GelDoc XR+ (Bio-Rad).

### Statistics

Data are presented as mean ± SD. Calculations were performed with Microsoft Excel.

### Data availability

Genetic sequencing and phenotype data utilized in this manuscript have been deposited in dbGaP, accession numbers phs001966 and phs001968. Please see dbGaP repositories for instructions on proper citation of this data. All REBELseq python scripts and reference files are available at GitHub (see link above). The University of Pennsylvania Institutional Review Board determined that this research qualified as Category IV exempt human research. Supplemental Material includes a representative bioanalyzer trace for a completed Ta subfamily L1Hs-enriched next generation sequencing library and the sequences of all oligomers used in this manuscript. Supplemental material available at figshare: https://doi.org/10.25387/g3.11894406.

## Results

### REBELseq identifies putative L1Hs retrotransposon insertions

**Figure 3:**
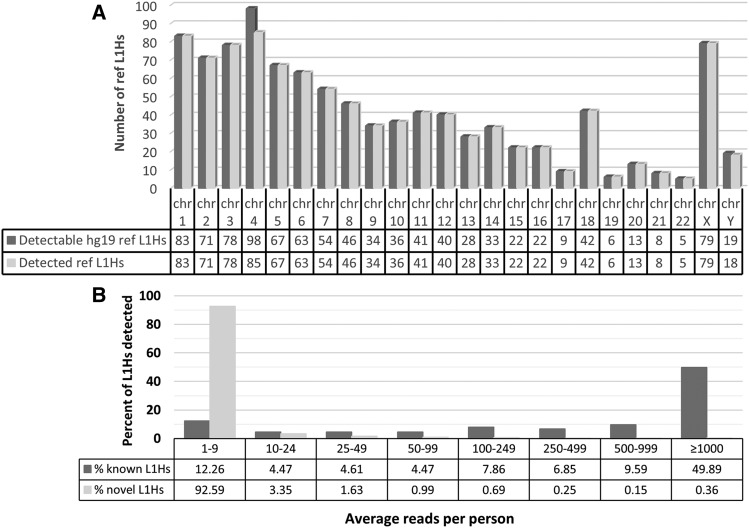
Detection levels of Ta subfamily L1Hs. (A) The number of Ta subfamily ref L1Hs detected per chromosome *vs.* the number of Ta subfamily ref L1Hs annotated in hg19 repeat masker. We detected ∼99% of Ta subfamily L1Hs annotated in hg19 repeat masker. (B) Average number of sequencing reads per person for a given L1 insertion. Data were binned to show contrast between the average number of sequencing reads per person seen for known and putative novel L1Hs insertions. An average number of sequencing reads per person ≥ 100 was used as a bioinformatic cutoff.

We performed REBELseq using DNA isolated from postmortem cortex NeuN+ nuclei samples of 177 individuals and identified a total of 157,178 independent putative Ta subfamily L1Hs insertions, with 1,050 annotating to hg19 known reference L1Hs elements (ref L1Hs) and 156,128 not annotating to hg19 known reference L1Hs (non-ref L1Hs). The ref L1Hs we detected represented ∼68% of all L1Hs (both Ta subfamily and non-Ta subfamily) annotated in hg19 repeat masker (1,050/1,544; data not shown). Given that REBELseq is specifically designed to identify only Ta subfamily L1Hs, we examined the number of hg19 repeat masker Ta subfamily ref L1Hs we identified, and we determined that we detected ∼99% (983/997; Figure 3A). This suggests that REBELseq identified non-Ta subfamily ref L1s at a rate of ∼6% of all putative ref L1s. Of the non-ref L1Hs, 432 annotated to known L1s in the 1000 genomes data (known non-ref L1Hs), while the other 155,696 are putative novel non-ref L1Hs. The REBELseq bioinformatics pipeline calculates the mean MapQ score, based on MapQ scores assigned by Bowtie2 during sequencing read alignments (see Methods), for each putative L1 insertion identified. The ref L1Hs we identified had an average mean MapQ of 38.82 ± 4.10. Having high confidence that the detected ref L1Hs were real, we used the ref L1Hs average mean MapQ minus two standard deviations (MapQ ≥ 30.62) as a bioinformatic cutoff of our data, after which we retained 974 ref L1Hs (92.76% of the 1,050 ref L1s), 430 known non-ref L1Hs (99.54% of 432) and 127,976 putative novel non-ref L1Hs (82.20% of 155,696).

In the remaining known (ref L1Hs and known non-ref L1Hs) and putative novel L1Hs, we next examined the average number of sequencing reads per person for a given L1Hs insertion (*i.e.*, the number of sequencing reads for the L1 insertion divided by the number of individuals which are reported as carriers for the L1 insertion; Figure 3B). A clear difference between the known and putative novel L1Hs emerged, with 92.6% of putative novel L1Hs having an average sequencing reads per person < 10, and 87.7% of known L1Hs having an average sequencing reads per person ≥ 10 and 74.2% having average reads per person ≥ 100. Having confidence in the known L1Hs pool, an average of ≥ 100 sequencing reads per person was used as a bioinformatic cutoff, which retained 699 ref L1Hs (66.57%), 332 known non-ref L1Hs (76.85%) and 1,842 putative novel non-ref L1Hs (1.18%).

### L1Hs insertion validation by PCR

**Figure 4:**
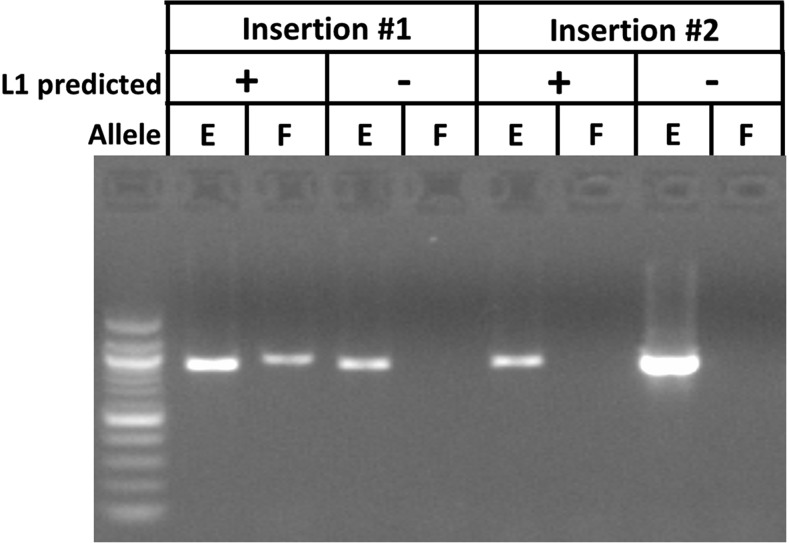
Example image of a successful and unsuccessful confirmatory PCR. PCR experiments were conducted to determine the proportion of putative novel non-ref L1Hs insertions detected by REBELseq that could be independently validated. For each insertion, gDNA from a person predicted to have the L1Hs insertion (+) and a person not predicted to have the insertion (-) were used to amplify the genomic region purported to contain the L1Hs insertion (Filled site, F) and the same genomic region if it did not contain the insertion (Empty site, E). Insertion #1 is a positive confirmation of the results predicted by REBELseq, while Insertion #2 is a negative confirmation.

Having established bioinformatic cutoffs for our data, we next sought to experimentally determine the proportion of bioinformatically-detected putative L1Hs insertions that were real using PCR of the 3′ insertion junction of the allele containing the insertion (filled allele) and the corresponding genomic region on the ‘empty’ allele (see Figure 4 for example image). Having confidence that the known L1Hs insertions, the ref L1Hs and known non-ref L1Hs, are real because they are annotated portions of the human reference genome or identified in the 1000 genomes sequencing project, respectively, we focused on the validation of the putative novel non-ref L1Hs insertions predicted by REBELseq (Table 1). The percentages of putative novel non-ref L1Hs insertions that could be validated by average read number bin were experimentally determined (% validated), and these data were used to calculate the predicted number of novel non-ref L1Hs insertions that we would expect to be true positives in each read bin (predicted true positive). Using the number of putative novel non-ref L1Hs insertions predicted to be true positives and the numbers of known L1Hs detected, we calculated the total true positive per read bin and the percentage true positive per read bin. We determined that ∼89% of detected L1Hs insertions having ≥1,000 average sequencing reads per person were true positives. Additionally, to better understand the relationship between the average number of sequence reads per person, which was used to define the bins, and the probability of an L1Hs insertion predicted by REBELseq to be a true positive, we calculated the cumulative percent true positive per bin, defined as all L1Hs insertions meeting the minimum average number of sequencing reads per person, or above, for that bin.

**Table 1:** Table describing the rates of L1Hs insertion validation by binned average number of sequencing reads. The percentage of successfully validated novel non-ref L1Hs per read bin was used to calculate the number of predicted true positive novel non-ref L1Hs per bin. The calculated total true positive per read bin is the sum of the predicted true positive novel non-ref L1Hs and the total number of detected known non-ref and ref L1Hs per bin. The calculated percent true positive per bin is calculated as the total true positive for bin, divided by the sum of the number of detected novel non-ref L1Hs, number of detected known non-ref L1Hs and number of detected ref L1Hs. The cumulative percent true positive is calculated as the percentage of true positive L1Hs insertions having at least the average number of sequencing reads for that bin and above

	novel non-ref L1Hs					known non-ref L1Hs	ref L1Hs	Calculated total true positive per bin	Calculated % true positive per bin	Cumulative % true positive (per bin and above)
Avg. # of reads	Successfully validated	Validations attempted	% Validated	total # detected per bin	Predicted true positive	total # detected per bin	total # detected per bin			
≥1,000	32	44	72.7	460	334	227	465	1026	89.1	89.1
500-999	16	41	39.0	186	73	43	90	206	64.6	83.8
250-499	13	40	32.5	320	104	28	67	199	48.0	75.9
100-249	6	36	16.7	876	146	34	77	257	26.0	58.8

Table 1. Validation of putative Ta subfamily L1Hs insertions.

### Distribution of detected L1Hs

**Figure 5:**
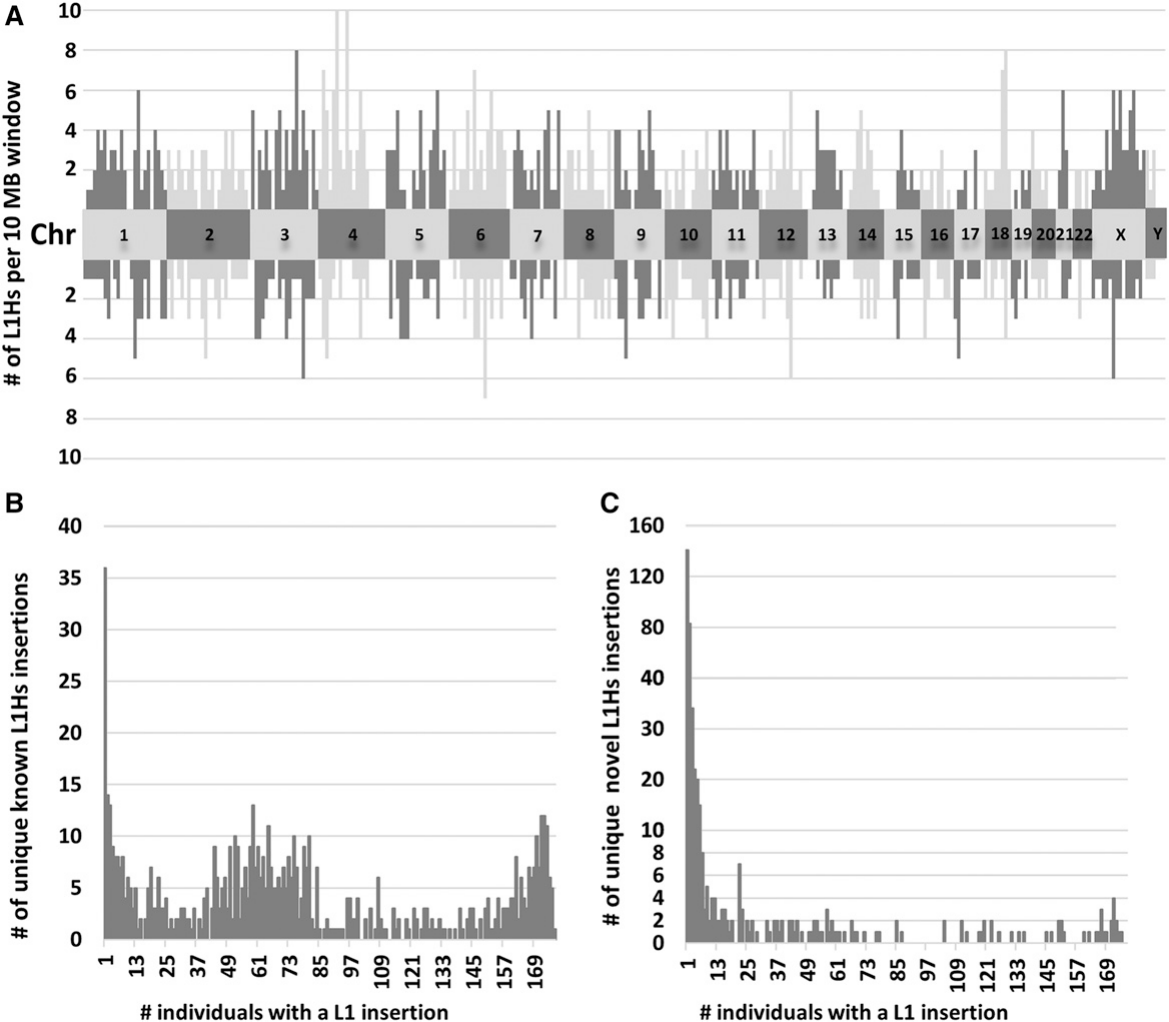
Distributions of high confidence L1Hs insertions. (A) The genomic distribution of known L1Hs (above the chromosome numbers) and novel L1Hs (below the chromosome numbers) per 10 MB window of each chromosome. Alternating color pattern and labeled central blocks represent the different chromosomes. Known and novel L1Hs are distributed throughout the genome, suggesting REBELseq is an unbiased whole genome approach. (B) Distribution of the number of unique known L1Hs insertions per a given number of individuals. The number of known L1Hs insertions shared by different numbers of individuals shows a trimodal distribution. While some known L1Hs occur with a rate of affected individuals approaching 1.0, the other local maxima are focused at few or less than half of surveyed individuals. This demonstrates that known L1Hs, such as those annotated in repeat masker for the human draft genome, should be considered polymorphic in nature, rather than ubiquitous in the human genome. (C) Distribution of the number of unique novel L1Hs insertions per a given number of individuals. The number of novel L1Hs insertions shared by different numbers of individuals shows a right skewed distribution. While some novel L1Hs were detected in the majority of individuals, most novel L1Hs were detected in one or a few individuals.

Having determined that most L1Hs insertions with ≥1,000 average sequencing reads per person were true positives (∼89%; see above), we sought to better understand the distribution of these high confidence L1Hs insertion across the genome and individuals. The genomic distribution of known L1Hs (above the chromosome numbers) and novel L1Hs (below the chromosome numbers), per 10MB genomic window, is depicted in Figure 5A, showing that these high confidence L1Hs are distributed throughout the genome. Finally, we determined the number of unique known L1Hs insertions (Figure 5B) and novel L1Hs insertions (Figure 5C) with a given number of individuals sharing that insertion.

## Discussion

While other methods for preparation of L1-targeted next generation sequencing libraries exist (Badge *et al.* 2003; Baillie *et al.* 2011; Costello *et al.* 2018; Erwin *et al.* 2016; Evrony *et al.* 2012; Ewing and Kazazian 2011; Iskow *et al.* 2010; Kvikstad *et al.* 2018; Streva *et al.* 2015; Upton *et al.* 2015), some are labor-intensive when scaled to a large number of individual genomes, because of reliance on gel purification (Ewing and Kazazian 2011; Streva *et al.* 2015) or because they require preparation of multiple amplicon libraries per individual sample, due to the use of multiple hemi-degenerate primers (Evrony *et al.* 2012; Ewing and Kazazian 2011). Some methods rely on the random shearing of DNA (Erwin *et al.* 2016; Upton *et al.* 2015; Zhao *et al.* 2019), a process that requires specialized equipment and somewhat abundant DNA quantity, while others may have reduced specificity for the Ta subfamily of L1Hs, due to primer design, which constitutes the majority of actively replicating L1 retrotransposons in the human genome (Baillie *et al.* 2011; Iskow *et al.* 2010; Kvikstad *et al.* 2018; Zhao *et al.* 2019). REBELseq was designed as a high throughput alternative method to reliably detect the 3′ flanking region of Ta subfamily L1Hs elements with limited gDNA input. It should be noted that REBELseq was not intended to determine whether an L1Hs insertion is full length or truncated or if ORF1 and ORF2 are intact, but merely to determine the presence or absence of the 3′ end of Ta subfamily L1Hs. After identification of insertions of interest, these other aspects can be determined by long range PCR and Sanger sequencing.

Other methods to produce L1-enriched libraries employing restriction enzyme digests of gDNA as a starting point have been described (Badge *et al.* 2003). There is no set requirement for which restriction enzyme to use in REBELseq other than that it generates blunt ends and cuts 5′ of the L1HsACA primer that identifies the 3′ end of the Ta subfamily L1Hs elements. REBELseq begins with restriction enzyme digestion of gDNA with HaeIII. This restriction enzyme was chosen for the following reasxons: 1) it creates blunt ends at 5′-GG|CC-3′ sequences such that polishing of cleaved ends is unnecessary; 2) it cuts the human genome to an average size of 342 ± 478 base pairs (NEBcutter) which is similar to the range of fragments generated by sonication; 3) it does not cut within the 3′ end of the L1Hs sequence targeted by our L1Hs Ta subfamily-specific primers. One possible concern of using a single restriction enzyme digestion in REBELseq library construction is the possibility that the restriction site for the enzyme occurs immediately downstream from a L1 insertion, thus preventing the single primer extension into the downstream gDNA (see Figure 1). While this raises the possibility that using a single restriction enzyme digestion during REBELseq library construction may not detect all L1 insertions, it does not reduce the validity of L1 insertion that are detected. One possible solution to this concern would be to perform restriction enzyme digestions with two or more enzymes that meet the above criteria and pool the fragments before the single primer extension step.

Our method is specifically designed to leverage diagnostic nucleotides specific to the Ta subfamily of L1Hs elements for differential amplification. It can easily be adapted to detect the 5′ flanking region of full length L1Hs elements in the human genome, although diagnostic nucleotides are limited within the 5′ end of L1Hs for the Ta subfamily. Reliable primers targeting the 5′ end of Ta subfamily L1Hs and an enzyme other than HaeIII would be required for analysis of full length L1Hs elements, as HaeIII makes a single digestion in the L1Hs sequence near the 5′ end. REBELseq could also be utilized to differentially amplify and identify evolutionarily older pre-Ta subfamily L1Hs insertions (by changing the last three nucleotides of the L1HsACA primer to ‘ACG’) or any type of L1Hs element (by eliminating the last nucleotide of the L1HsACA primer to end in ‘AC’). Additionally, this method could be utilized to amplify and identify almost any other genomic element with a specific pair of nested primers (to replace L1HsACA and L1HsG).

The REBELseq technique is compatible with human gDNA purified from any tissue source, including gDNA purified from leukocytes in saliva and blood. In the application of REBELseq presented in this manuscript, 177 fresh frozen human postmortem prefrontal cortex brain samples were utilized for purification of gDNA from NeuN+ neuronal nuclei. Utilizing gDNA from NeuN+ neuronal nuclei allows for the identification of germline L1s, similar to using gDNA from a peripheral source, and individual neuronally relevant somatic mutations occurring: A) only in the brain (an L1 that retrotransposed early in neuronal development), B) in a limited number of neurons (an L1 that retrotransposed in a neuronal progenitor cell, producing a cluster of neurons harboring the insertion), or C) in a single neuron (an L1 that retrotransposed in the neuron post-mitotically), results which could be verified by comparing the REBELseq results from NeuN+ nuclei to those of a peripheral tissue. Using REBELseq to compare Ta subfamily L1Hs from multiple brain regions would also allow for the identification of regionally specific L1Hs insertions, possibly relevant in the context of neurological and psychiatric disease.

Using REBELseq, before bioinformatics cutoffs, we identified 157,178 independent putative Ta subfamily L1Hs insertions, with 1,050 annotating to reference L1Hs in hg19 repeat masker. The ref L1Hs detected represent ∼68% of all hg19 repeat masker L1Hs and ∼99% of Ta subfamily L1Hs that we had expected to detect (Figure 3A). The bioinformatic cutoffs described in this manuscript were empirically derived by the average mean MapQ score for the ref L1Hs, the average number of sequencing reads per individual per insertion (Figure 3B), and experimental confirmation of putative novel non-ref L1Hs insertions (Table 1). We were unable to experimentally validate all putative novel L1Hs in any of our average read bins. This is possibly due to the detected L1Hs insertion being sequencing reads of chimeric PCR products, or possibly because Ta subfamily L1Hs frequently retrotranspose into repetitive sequences in the genome (*e.g.*, the remnants of older repetitive elements), making both their genomic alignment and PCR validation difficult. We believe this suggests the proportion we were able to validate likely represents a minimum value, with additional detected insertions being confirmable with more complex PCR methods. ∼70% of all known L1Hs averaged at least 100 sequencing reads per person, with our experimentally determined probability of validating any detected L1Hs above 100 reads being ∼59% (cumulative % true positive, Table 1). Examining the data with ≥1,000 average sequencing reads per person, we still see ∼47% of all detected known L1Hs and a probability of validating any detected L1Hs of ∼89%. These data suggest that novel non-ref L1Hs having ≥1,000 reads per person include what are likely real polymorphic and somatic L1Hs insertions. As an alternative to our method of bioinformatically identifying putative Ta subfamily L1Hs insertions from our sequencing data, it may be possible to utilize a machine learning approach, similar to that of Tang *et al.* (2017), to identify a pool of putative Ta subfamily L1Hs insertions that would later have a higher chance of being experimentally validated.

When examining the genomic distribution of known and novel L1Hs having an average of ≥1,000 sequencing reads per person (Figure 5A), we see that both the known and novel L1Hs are dispersed throughout the genome, suggesting that REBELseq is an unbiased whole genome approach. Assessing the distribution of the number of individuals (ranging from 1-177) having either a known (Figure 5B) or novel (Figure 5C) L1Hs insertion, we observe that both groups have L1Hs insertions occurring throughout the range of possible values (*i.e.*, the number of individuals sharing a given insertion). With respect to the known L1Hs (Figure 5B), there appears to be a trimodal distribution with ∼37% of L1Hs occurring in 45-85 individuals and ∼9% occurring in ≤ 3 and ≥ 170. This means that ∼55% of insertions occur in only ∼29% of the possible numbers of individuals sharing a given insertion. This finding, that a large proportion of known L1Hs insertions occur in a small fraction of individuals, disagrees with a previous report (Ewing and Kazazian 2011), but we believe this may be due to their small sample size, thereby diminishing the likelihood that an individual would not have a given insertion. ∼70% of novel L1Hs occur in ≤ 7 people, suggesting that these L1 insertions are either lower minor allele frequency events or individual somatic insertions. ∼8% of novel L1Hs occur in more than two thirds of individuals, suggesting that these L1Hs insertions likely represent ref L1Hs occurring at common minor allele frequencies that are not cataloged in hg19 repeat masker.

Taken together, we believe these data demonstrate that REBELseq reliably detects both known and novel Ta subfamily L1Hs insertions, and that this technique could be a powerful method for examining the association of the prevalence of L1Hs insertions in a broad range of human disease.
